# Association of dietary fiber intake with current asthma and recent asthma outcomes: a cross-sectional analysis of National Health and Nutrition Examination Survey 2017–March 2020 pre-pandemic data

**DOI:** 10.29219/fnr.v70.14782

**Published:** 2026-10-23

**Authors:** Tingting Zhao

**Affiliations:** Department of Respiratory and Critical Care Medicine, Zhuji People’s Hospital, Zhejiang, China

**Keywords:** dietary fiber, asthma, fatty acids volatile, cross-sectional studies, nutrition surveys

## Abstract

**Objective:**

Dietary fiber may influence asthma through the gut-lung axis and systemic inflammation, but evidence in contemporary U.S. adults remains limited.

**Methods:**

We analyzed 6,145 adults aged 20 years or older from National Health and Nutrition Examination Survey 2017–March 2020 pre-pandemic data using a common complete-case sample for the staged regression models. Day-1 dietary fiber was the primary exposure; a separate 2-day mean sensitivity analysis was conducted in 5,364 participants with a reliable second recall. Survey-weighted logistic regression assessed current asthma, asthma attacks and asthma-related acute-care visits with adjustment for demographic, socioeconomic, lifestyle, energy-intake and clinical covariates; body mass index (BMI) was evaluated separately. False-discovery-rate correction across the three outcomes and multiple imputation were used as sensitivity analyses.

**Results:**

Current asthma was present in 596 participants (weighted prevalence, 8.7%). Each 1-standard-deviation increase in Day-1 fiber was associated with lower odds of current asthma in the primary model (odds ratio [OR], 0.80; 95% confidence interval [CI], 0.64–0.99; *P* = 0.043), but not after false-discovery-rate correction (*q* = 0.129). BMI adjustment yielded OR 0.82 (95% CI, 0.66–1.02; *P* = 0.076). Separate repeated-recall and multiple-imputation analyses were directionally consistent (2-day mean: OR, 0.74; 95% CI, 0.61–0.90; multiple imputation: OR, 0.81; 95% CI, 0.66–0.99). Fiber was not associated with asthma attacks or acute-care visits.

**Conclusion:**

Higher reported fiber intake showed an inverse association with current asthma, but statistical evidence was modest after multiple-outcome correction. Repeated-recall and missing-data analyses were directionally consistent. Cross-sectional design, reverse causation and residual confounding preclude causal interpretation.

## Popular scientific summary

- Diet quality and gut microbiota-related metabolites have been linked to asthma.
- Higher dietary fiber intake was associated with lower odds of current asthma.
- The findings support reinforcing dietary fiber as a practical target in population nutrition strategies.

Asthma is one of the most common chronic respiratory diseases worldwide and imposes a substantial burden of morbidity, healthcare use and premature death. The Global Burden of Disease Study 2021 estimated that asthma affected more than 260 million people and ranked it among the leading causes of disability from chronic respiratory illness, with a disease burden shaped by modifiable risk factors including high body mass index (BMI) and smoking (1). In the United States, current asthma affects approximately 8.9% of adults, or nearly one in 11 adults, and many patients continue to experience exacerbations, acute-care visits and impaired quality of life despite available inhaled therapies (2). Because airway and systemic inflammation are influenced by lifestyle, there is continuing interest in identifying modifiable, non-pharmacological exposures, particularly diet that may be related to asthma prevalence or severity (3).

Dietary fiber is a leading candidate exposure. Fiber comprises non-digestible plant carbohydrates that reach the colon largely intact; the fermentable fraction is metabolized by the gut microbiota to short-chain fatty acids such as acetate, propionate and butyrate. These metabolites nourish the colonic epithelium, reinforce the intestinal barrier and exert systemic immunoregulatory effects, providing a biological rationale for a protective relationship with inflammatory disease (4, 5). Despite these recognized benefits, fiber intake in the United States is low: mean adult consumption is approximately 17 g/day, only about half of the recommended adequate intake, and more than 90% of women and 97% of men fall short of guideline targets (6). This pervasive shortfall makes the fiber-asthma relationship relevant for public-health research.

A growing body of evidence links the gut microbiome to airway health through a bidirectional gut-lung axis, in which microbial composition and microbial metabolites influence pulmonary immune responses (7, 8). Fermentable fiber increases short-chain fatty-acid production, which can promote regulatory T-cell differentiation and function, restrain type-2 innate lymphoid-cell and T-helper-2 responses, and dampen pro-inflammatory cytokine output through G-protein-coupled-receptor signaling and histone-deacetylase inhibition; butyrate in particular has been shown experimentally to attenuate allergic airway inflammation and airway hyperreactivity (9–11). These mechanisms suggest a pathway through which diet could be related to asthma both directly, through immune modulation, and indirectly, through adiposity and metabolic health, given the established relationship between obesity and adult asthma (12, 13).

Epidemiological studies have connected dietary quality to asthma, but much of the evidence concerns broad dietary indices rather than fiber itself. Cross-sectional analyses of the National Health and Nutrition Examination Survey (NHANES) have linked higher total energy intake, a more pro-inflammatory diet as captured by the dietary inflammatory index, lower diet quality on the Healthy Eating Index and higher consumption of ultra-processed foods to greater asthma prevalence, whereas minimally processed foods appear protective (14–17). Systematic-review and case-control evidence also supports inverse associations for fruit-and-vegetable-rich or Mediterranean-style dietary patterns, and Mendelian randomization analyses have examined specific dietary factors in relation to asthma susceptibility (18–20). Most directly, a previous NHANES analysis reported that higher dietary fiber intake was associated with lower prevalence of chronic inflammatory airway disease, including approximately 29% lower odds of asthma in the highest versus lowest fiber category (21). However, that work combined heterogeneous airway diseases, and few studies have focused specifically on asthma in the May 2017–March 2020 pre-pandemic NHANES file or examined whether any fiber-asthma association extends to acute disease events such as attacks and acute-care visits.

We therefore used nationally representative NHANES 2017–March 2020 pre-pandemic data to examine the association between dietary fiber intake and asthma outcomes among U.S. adults. We hypothesized that higher fiber intake would be associated with lower odds of current asthma, and we additionally evaluated two markers of asthma activity-recent asthma attacks and asthma-related acute-care visits. To strengthen interpretation within the constraints of a cross-sectional design, we modeled fiber continuously and in quartiles, characterized the dose-response with restricted cubic splines, evaluated repeated dietary recalls, assessed the impact of missing covariate data and multiple outcome testing, examined the conceptual role of BMI separately from the core confounder set, and tested the stability of findings in prespecified subgroup and sensitivity analyses. Throughout, results are framed as associations rather than causal effects.

## Methods

### Study design, data source and study population

This was a cross-sectional analysis of the NHANES, a continuous, nationally representative program conducted by the National Center for Health Statistics (NCHS) of the U.S. Centers for Disease Control and Prevention. NHANES characterizes the health and nutritional status of the civilian, non-institutionalized U.S. population using a complex, multistage probability sampling design and combines an in-home interview with a standardized examination in a mobile examination center. Because field operations for the 2019–2020 cycle were suspended owing to the COVID-19 pandemic, the partially completed May 2019 to March 2020 data were combined with the complete 2017–2018 cycle and released as the nationally representative May 2017 to March 2020 pre-pandemic data file (22). The NCHS Research Ethics Review Board approved the survey protocol and all participants provided written informed consent; because this secondary analysis used de-identified public-use data, additional institutional review was not required.

From the 15,560 participants in the 2017 to March 2020 file, we restricted the sample to the 9,232 adults aged 20 years or older, the target population for this adult analysis. We then required a reliable and complete Day-1 dietary recall with a non-missing dietary fiber value and valid Day-1 dietary sampling weight, plausible total energy intake (500–3,500 kcal/day in women and 500–4,500 kcal/day in men), and non-missing current-asthma status. These criteria yielded 7,302 otherwise eligible adults. For direct comparison across the staged complete-case models, we used a common analytic sample with complete model covariates and measured BMI, yielding 6,145 adults. BMI availability was required to keep Models 1–4 on the same participants; BMI itself was not included in the primary confounder-adjusted Model 3. This common-sample definition excluded 91 otherwise eligible adults solely because BMI was missing. Potential bias related to missing data and the common-sample restriction was assessed using participant comparisons and multiple imputation. Participant selection is summarized in Fig. 1. After incorporation of the Day-1 dietary sampling weights, the common complete-case sample represented approximately 199.4 million non-institutionalized U.S. adults.

**Fig. 1:**
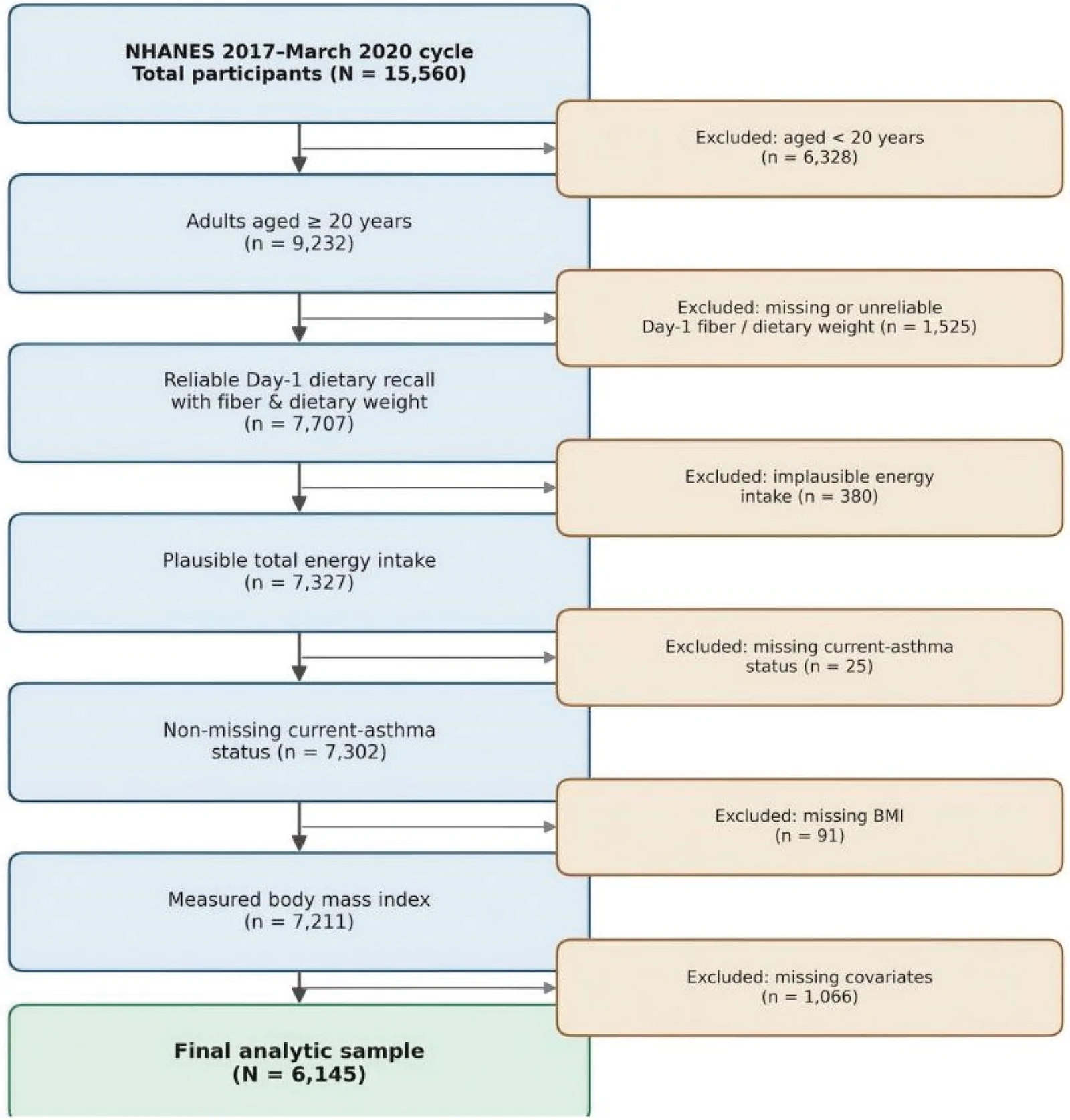
Flow diagram of participant selection. Beginning with the full NHANES 2017–March 2020 sample, participants were restricted to adults aged ≥20 years and then sequentially excluded for missing or unreliable dietary fiber data, implausible energy intake, missing current-asthma status, missing BMI, and missing covariates, yielding the common complete-case sample used for Models 1–4 (*N* = 6,145). BMI availability was required to keep the staged models on the same participants; BMI was not included as a covariate in the primary Model 3. BMI: body mass index; NHANES: National Health and Nutrition Examination Survey.

### Assessment of dietary fiber intake (exposure)

Dietary intake in NHANES is ascertained using the U.S. Department of Agriculture Automated Multiple-Pass Method, a validated, computer-assisted 24-h dietary recall that systematically prompts respondents to report all foods and beverages consumed in the preceding day. The first recall (Day 1) is administered in person in the mobile examination center, and total dietary fiber (g/day) is derived from the Total Nutrient Intakes file by linking reported foods to the Food and Nutrient Database for Dietary Studies (23). Day-1 total dietary fiber was the primary exposure because the Day-1 sample is larger and is paired with the appropriate pre-pandemic Day-1 dietary weight (WTDRD1PP). The exposure was analyzed continuously per 1-standard-deviation increment, in sample-based quartiles with the lowest quartile as the referent and a quartile-rank trend term, and flexibly using restricted cubic splines. In the primary sample, mean Day-1 fiber intake was 16.2 g/day (standard deviation 9.8), the median was 13.8 g/day, and quartile cut-points were approximately 8.9, 13.8 and 20.4 g/day. Among the 6,145 participants in the primary analysis, 5,364 (87.3%) also had a reliable second 24-h recall available in the NHANES second-day nutrient file (24). For a repeated-recall sensitivity analysis, we calculated the mean of Day-1 and Day-2 fiber intake and analyzed it with the corresponding 2-day dietary weight (WTDR2DPP). Averaging two recalls can reduce the influence of day-to-day variation, but the repeated-recall analysis used a smaller participant subset, a different dietary weight and a different exposure standard deviation; its odds-ratio magnitude was therefore not interpreted as directly comparable with the Day-1 estimate or as a formal estimate of long-term usual intake. Fiber density expressed per 1,000 kcal was evaluated in an additional sensitivity analysis.

### Asthma outcome definitions

Asthma outcomes were defined from the NHANES Medical Conditions questionnaire (25). The primary outcome, current asthma, was defined as an affirmative response to whether a doctor or other health professional had ever told the participant that they had asthma (MCQ010), followed by an affirmative response to whether they still had asthma (MCQ035). Participants who reported never having had asthma were classified as not having current asthma, and those with indeterminate responses were excluded. Two secondary outcomes captured recent asthma activity as population-level indicators: (1) an asthma attack, defined as reporting an asthma episode or attack in the previous 12 months (MCQ040); and (2) an asthma-related acute-care visit, defined as reporting an emergency-department or urgent-care visit for asthma in the previous 12 months (MCQ050). Because MCQ040 and MCQ050 are administered only to participants who report current asthma, individuals without current asthma were coded as not having these events in the primary population-level analyses. To check whether this coding choice affected interpretation, we also re-estimated the attack association among participants with a history of asthma. In the final sample, current asthma was present in 596 participants, an asthma attack in 262, and an asthma-related acute-care visit in 111.

### Covariates

Covariates were selected a priori as potential common causes of dietary fiber intake and asthma on the basis of biological plausibility, prior literature and a directed-acyclic-graph-based conceptual framework (26). The core confounder set comprised demographic and socioeconomic factors (age, sex, race and ethnicity, educational attainment and family income-to-poverty ratio), lifestyle factors (cigarette smoking, alcohol consumption, physical activity and total daily energy intake), and clinical comorbidities (physician-diagnosed hypertension and diabetes). BMI, computed from measured weight and height, was treated separately because its causal role is ambiguous: socioeconomic and lifestyle factors may influence both fiber intake and asthma, whereas fiber intake may also influence adiposity, which in turn is associated with asthma. Accordingly, BMI was excluded from the primary confounder-adjusted model and included in a separate sensitivity model; this BMI-adjusted estimate was not interpreted as a definitive controlled direct effect. For descriptive and mechanistic context, waist circumference, the complete blood count-derived neutrophil-to-lymphocyte ratio, systemic immune-inflammation index and high-sensitivity C-reactive protein were also tabulated.

### Statistical analysis

All analyses incorporated the NHANES complex survey design using the appropriate pre-pandemic dietary sampling weights together with masked sampling strata (SDMVSTRA) and primary sampling units (SDMVPSU). Variances were estimated by the design-based Taylor-linearization method so that estimates and confidence intervals [CIs] generalize to the U.S. adult population represented by the May 2017–March 2020 pre-pandemic dietary sample. Weighted baseline characteristics were summarized overall and across fiber quartiles as weighted means with standard deviations for continuous variables and weighted percentages for categorical variables, with differences across quartiles evaluated by design-based tests (Table 1).

**Table 1:** Weighted characteristics of the analytic sample by quartile of Day-1 dietary fiber intake

Characteristic	Overall	Quartile 1	Quartile 2	Quartile 3	Quartile 4	*P*
No. of participants	6,145	1,541	1,532	1,545	1,527	-
Asthma outcomes, weighted %						
Current asthma	8.7	11.6	8.2	8.0	7.2	0.002
Asthma attack, past 12 mo	3.9	4.2	3.9	3.5	4.0	0.24
Asthma-related acute-care visit	1.3	1.8	1.6	1.1	1.0	0.13
Continuous variables, weighted mean (SD)						
Age, years	48.7 (17.4)	47.3 (17.3)	48.6 (18.0)	49.0 (17.4)	49.5 (16.8)	0.06
Dietary fiber, g/day	16.2 (9.8)	6.1 (2.0)	11.4 (1.3)	16.8 (1.9)	29.2 (8.5)	<0.001
Total energy, kcal/day	2,064 (797)	1,496 (588)	1,912 (665)	2,233 (707)	2,549 (798)	<0.001
BMI, kg/m^2^	29.8 (7.1)	30.5 (7.2)	30.1 (7.4)	29.7 (7.2)	29.0 (6.6)	<0.001
Waist circumference, cm	100.8 (16.8)	101.8 (17.0)	101.4 (17.2)	100.6 (16.9)	99.3 (16.1)	0.003
Family income-to-poverty ratio	3.2 (1.6)	2.9 (1.7)	3.1 (1.6)	3.3 (1.6)	3.4 (1.6)	<0.001
Physical activity, MET-min/week	4,570 (6,812)	5,201 (7,521)	4,495 (7,156)	3,975 (5,977)	4,686 (6,566)	0.25
Neutrophil-to-lymphocyte ratio	2.2 (1.1)	2.2 (1.3)	2.2 (1.2)	2.1 (1.1)	2.2 (1.0)	0.32
Systemic immune-inflammation index	532 (317)	557 (337)	544 (345)	515 (288)	517 (296)	<0.001
hs-CRP, mg/L	3.7 (6.6)	4.4 (8.1)	3.5 (5.4)	3.6 (5.7)	3.2 (6.8)	0.005
Categorical variables, weighted %						
Female sex	51.6	59.7	55.7	48.4	43.8	<0.001
Physically active (≥600 MET-min/wk)	68.8	66.7	64.6	66.5	77.0	<0.001
Current alcohol drinker	93.5	94.9	93.8	93.3	92.2	0.12
Hypertension	33.4	36.1	33.5	34.8	29.4	0.009
Diabetes	11.6	12.1	11.8	12.1	10.6	0.04
Obese (BMI ≥30 kg/m^2^)	42.8	49.9	44.1	39.3	38.8	<0.001
Income-to-poverty ratio ≥3.5	47.5	41.3	44.0	51.1	52.8	<0.001
Race / ethnicity, weighted % (*P* < 0.001)						
Non-Hispanic White	64.8	64.6	64.7	68.9	60.7	
Non-Hispanic Black	10.6	14.9	11.3	9.6	7.0	
Hispanic	14.9	11.8	13.8	12.6	21.3	
Non-Hispanic Asian	5.6	3.5	4.7	5.5	8.4	
Other / multiracial	4.1	5.2	5.5	3.4	2.6	
Education, weighted % (*P* < 0.001)						
Less than high school	9.4	11.2	9.3	7.1	10.3	
High-school graduate	27.6	36.0	29.3	25.6	20.4	
Some college or above	63.0	52.8	61.5	67.2	69.2	
Smoking status, weighted % (*P* < 0.001)						
Never smoker	57.6	49.6	56.3	61.2	62.4	
Former smoker	26.2	23.3	27.4	26.2	27.8	
Current smoker	16.2	27.1	16.3	12.6	9.8	

Note: Continuous variables are weighted means (standard deviation); categorical variables are weighted percentages. *P*-values are from design-based tests across quartiles. Quartile cut-points were approximately 8.9, 13.8 and 20.4 g/day. BMI: body mass index; hs-CRP: high-sensitivity C-reactive protein; MET: metabolic equivalent.

The primary analyses used survey-weighted logistic regression of each binary asthma outcome on dietary fiber, estimated under a progressively adjusted modeling sequence. Model 1 was unadjusted. Model 2 adjusted for age, sex, race and ethnicity, education and income. Model 3, the primary confounder-adjusted model, additionally adjusted for smoking, alcohol use, physical activity, total energy intake, hypertension and diabetes, but did not include BMI. Model 4 added BMI to Model 3 as a sensitivity analysis to quantify attenuation when adiposity was included. To permit direct comparison of coefficients across these nested stages, Models 1–4 were fit in the same common complete-case sample (*N* = 6,145), which required BMI availability for Model 4 even though BMI was not a covariate in Model 3. Each outcome was modeled both per 1-standard-deviation increment in fiber and across fiber quartiles, with a linear trend tested by modeling quartile rank as an ordinal term. Results are expressed as OR with 95% CI (Table 2).

**Table 2:** Survey-weighted associations between Day-1 dietary fiber intake and asthma outcomes

Model/contrast	OR (95% CI)	*P*
Current asthma		
Model 1 (crude)	0.78 (0.65, 0.93)	0.006
Model 2	0.88 (0.73, 1.06)	0.156
Model 3 (main)	0.80 (0.64, 0.99)	0.043
Model 4 (+ BMI)	0.82 (0.66, 1.02)	0.076
Quartile 1 (reference)	1.00	-
Quartile 2	0.65 (0.42, 1.02)	0.062
Quartile 3	0.67 (0.45, 1.00)	0.050
Quartile 4	0.63 (0.39, 1.01)	0.057
*P for trend*		0.067
Asthma attack (past 12 months)		
Model 1 (crude)	0.88 (0.70, 1.11)	0.263
Model 2	0.98 (0.76, 1.27)	0.888
Model 3 (main)	0.90 (0.64, 1.26)	0.523
Model 4 (+ BMI)	0.94 (0.67, 1.32)	0.731
Quartile 1 (reference)	1.00	-
Quartile 2	0.89 (0.47, 1.66)	0.697
Quartile 3	0.86 (0.45, 1.64)	0.642
Quartile 4	1.04 (0.43, 2.51)	0.930
*P for trend*		0.939
Asthma-related acute-care visit		
Model 1 (crude)	0.74 (0.46, 1.17)	0.190
Model 2	0.90 (0.56, 1.44)	0.649
Model 3 (main)	0.89 (0.53, 1.48)	0.631
Model 4 (+ BMI)	0.92 (0.55, 1.56)	0.760
Quartile 1 (reference)	1.00	-
Quartile 2	0.95 (0.31, 2.92)	0.920
Quartile 3	0.84 (0.19, 3.74)	0.813
Quartile 4	0.83 (0.23, 2.99)	0.773
*P for trend*		0.755

Note: Per 1-standard-deviation estimates (approximately 9.8 g/day) are shown for four models; quartile contrasts and trend tests are from Model 3. Model 1, crude; Model 2, adjusted for age, sex, race/ethnicity, education and income; Model 3, the primary confounder-adjusted model, additionally adjusted for smoking, alcohol, physical activity, total energy, hypertension and diabetes; Model 4, Model 3 plus body mass index (BMI) as a sensitivity analysis. False-discovery-rate-adjusted *q* values across the three prespecified Model 3 outcomes are reported in Supplementary Table 6. OR: odds ratio; CI: confidence intervals.

Secondary and sensitivity analyses were specified before the final models were interpreted. Dietary fiber was modeled with restricted cubic splines using three knots at the 10th, 50th and 90th percentiles, with the median (13.8 g/day) as the reference; departure from linearity was assessed by testing the non-linear spline term. Effect modification of the fiber-current-asthma association was examined in prespecified subgroups defined by sex, age group, BMI category and smoking status, with multiplicative interaction tests. Sensitivity analyses evaluated the 2-day mean fiber exposure, fiber density per 1,000 kcal, additional BMI adjustment, exclusion of current smokers, exclusion of participants with obesity, and the attack association among participants with a history of asthma. To address multiplicity across the three prespecified asthma outcomes, Benjamini-Hochberg false-discovery-rate correction was applied to the Model 3 outcome-specific *P*-values (27). The progressively adjusted stages of the same outcome model were not treated as separate hypothesis families. Two-sided nominal *P*-values are reported throughout; primary-outcome findings are interpreted in conjunction with the false-discovery-rate-adjusted *q* value, while subgroup and additional sensitivity analyses are considered exploratory.

Missing covariate data were handled by complete-case analysis in the primary modeling sequence. The common complete-case sample was defined as participants with non-missing values for all covariates needed across Models 1–4, including BMI, so that staged estimates could be compared in the same participants. Because BMI was not included in the primary Model 3, this common-sample decision excluded 91 otherwise eligible adults solely because BMI was unavailable; we therefore explicitly considered possible selection and loss of precision from this restriction. Participants retained in the common complete-case sample were compared with otherwise eligible participants excluded because of missing BMI or model covariates. Missing covariates were additionally imputed using multiple imputation by chained equations with 20 imputed datasets; the imputation model included dietary fiber, asthma status, all analysis covariates, survey design variables and auxiliary demographic variables, and estimates were combined using Rubin’s rules (28). The imputed analyses used all 7,302 otherwise eligible adults and were used to assess the sensitivity of the primary association to complete-case selection. As a sensitivity analysis for residual unmeasured confounding, *E*-values were calculated for the Model 3 current-asthma point estimate and the confidence limit closest to the null (29). Analyses were implemented in Python (version 3), and reproducible code is available from the corresponding author on reasonable request.

## Results

### Participant selection and weighted characteristics

The derivation of the analytic sample is summarized in Fig. 1. Of the 15,560 participants in the NHANES 2017–March 2020 file, 9,232 were aged 20 years or older. Successive exclusions removed 1,525 participants without a reliable Day-1 recall or fiber value, 380 with implausible energy intake and 25 with missing current-asthma status, leaving 7,302 otherwise eligible adults. Of these, 91 (1.2%) lacked BMI and 1,066 of the remaining 7,211 had at least one missing model covariate, predominantly the family income-to-poverty ratio. To keep Models 1–4 on a common complete-case sample for direct comparison, BMI availability was required even though BMI was not included in the primary Model 3. Thus, 6,145 of 7,302 otherwise eligible adults (84.2%) were included in the common complete-case analysis and 1,157 (15.8%) were excluded because of missing BMI or model covariates. Participants excluded for missing data had slightly lower mean fiber intake and educational attainment and were more likely to be current smokers, while weighted current-asthma prevalence was similar to that in complete cases (9.4% vs. 8.7%) (Supplementary Tables 1 and 2). Applying the dietary sampling weights, the common complete-case sample represented approximately 199.4 million non-institutionalized U.S. adults.

Weighted characteristics of the sample, overall and by fiber quartile, are presented in Table 1. The weighted mean age was 48.7 years and 51.6% were women. Mean Day-1 dietary fiber intake was 16.2 g/day, ranging from 6.1 g/day in the lowest quartile to 29.2 g/day in the highest quartile and remaining below the recommended adult adequate-intake range of approximately 22–34 g/day. Higher fiber intake was associated with a more favorable risk-factor profile: participants in the upper quartiles were more likely to have attained at least some college education, to report higher family income, to be physically active and to be never-smokers, and they had higher total energy intake. The distribution of race and ethnicity also differed across quartiles. Higher fiber intake was accompanied by lower BMI and waist circumference, lower prevalence of hypertension and diabetes, and lower systemic immune-inflammation index and high-sensitivity C-reactive protein; the neutrophil-to-lymphocyte ratio did not differ materially across quartiles. The weighted prevalence of current asthma decreased across fiber quartiles, from 11.6% in the lowest to 7.2% in the highest (*P* = 0.002), whereas the prevalence of recent asthma attacks was essentially flat (*P* = 0.24) and that of acute-care visits declined without reaching statistical significance (*P* = 0.13).

### Association of dietary fiber with asthma outcomes

Survey-weighted associations between dietary fiber and each asthma outcome are reported in Table 2. For current asthma, each 1-standard-deviation increment in Day-1 fiber (approximately 9.8 g/day) was associated with lower odds in the unadjusted model (odds ratio [OR] 0.78, 95% CI 0.65–0.93; *P* = 0.006). The estimate was attenuated after demographic and socioeconomic adjustment (Model 2: OR 0.88, 95% CI 0.73–1.06; *P* = 0.156) and was nominally significant in the primary confounder-adjusted Model 3 (OR 0.80, 95% CI 0.64–0.99; *P* = 0.043). After Benjamini-Hochberg correction across the three prespecified Model 3 asthma outcomes, the current-asthma association did not remain statistically significant (*q* = 0.129); the corresponding q values were 0.631 for asthma attacks and 0.631 for acute-care visits. When BMI was added in Model 4, the estimate was OR 0.82 (95% CI 0.66–1.02; *P* = 0.076). In quartile analyses, participants in the highest fiber quartile had lower odds of current asthma than those in the lowest quartile (OR 0.63, 95% CI 0.39–1.01), with intermediate quartiles also below unity and a borderline linear trend (*P* for trend = 0.067).

By contrast, dietary fiber was not associated with either marker of acute asthma activity. For asthma attacks in the previous 12 months, point estimates were close to the null across all models (Model 3 per 1-standard-deviation: OR 0.90, 95% CI 0.64–1.26; *P* = 0.52), with no quartile gradient and a flat trend (*P* for trend = 0.94). For asthma-related acute-care visits, the per-standard-deviation point estimate was below unity but imprecise and non-significant (Model 3: OR 0.89, 95% CI 0.53–1.48; *P* = 0.63), reflecting the small number of events (111). The distributions of Day-1 fiber intake across outcome groups are displayed in Fig. 2: the median fiber intake was lower in participants with current asthma than in those without (12.7 vs. 13.9 g/day), whereas distributions were more similar for attacks and acute-care visits.

**Fig. 2:**
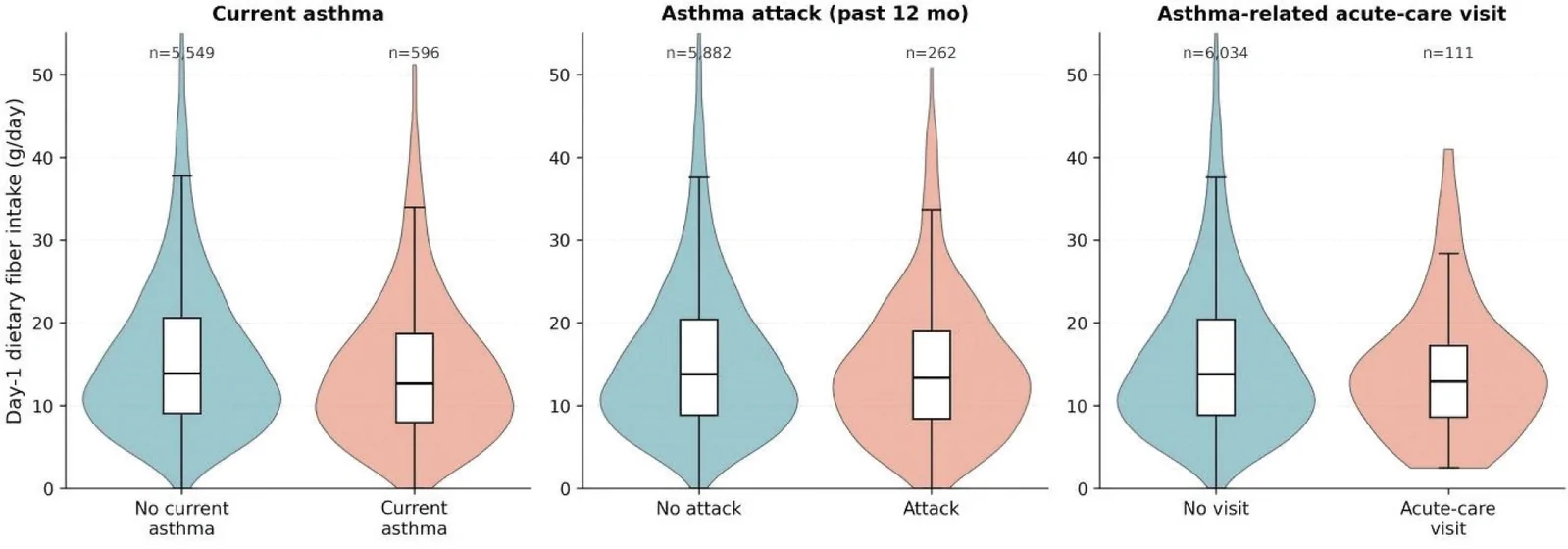
Distribution of Day-1 dietary fiber intake by asthma outcome status. Violin plots show the full kernel-density distribution of fiber intake within each group; the inner box marks the interquartile range and the horizontal line the median. Participants with current asthma had a lower median fiber intake than those without, whereas the distributions differed little by asthma-attack or acute-care-visit status. Group sample sizes are annotated.

### Dose–response relationship

Restricted cubic spline models, fitted with full covariate adjustment, are shown in Fig. 3. For current asthma, the adjusted OR described an approximately monotonic decline across the observed range of intake, falling from above unity at low fiber intakes to roughly 0.80 at higher intakes relative to the median. The formal test for non-linearity was not significant (*P* = 0.08), indicating that the inverse association was reasonably approximated by a log-linear relationship, although with a suggestion of a steeper gradient at the lowest intakes. The dose-response curves for asthma attacks and acute-care visits were comparatively flat and centered near the null across the range of fiber intake, and tests for non-linearity were non-significant for both outcomes (*P* = 0.70 and *P* = 0.71, respectively). The spline analysis was therefore consistent with an inverse relationship between fiber and current asthma prevalence, but not with a discernible dose-response for acute disease events.

**Fig. 3:**
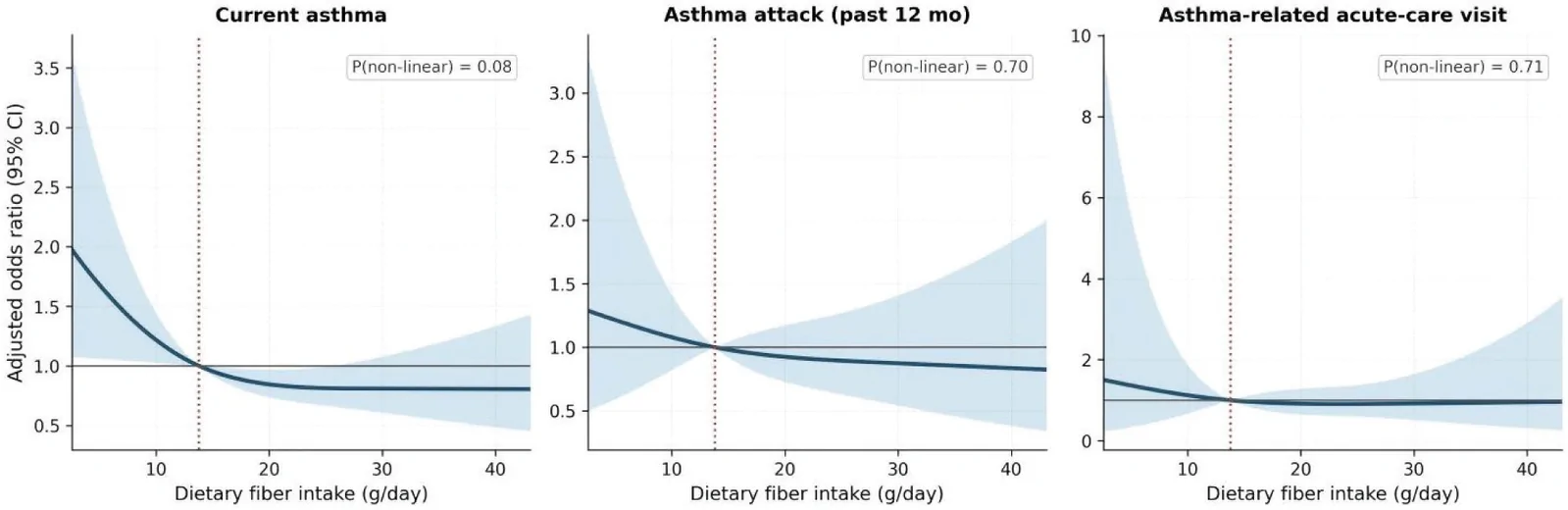
Dose-response associations between dietary fiber intake and asthma outcomes from restricted cubic spline models. Curves are adjusted odds ratios relative to the median fiber intake (13.8 g/day; red dotted line), with knots at the 10th, 50th and 90th percentiles. Models are fully adjusted (Model 3) and incorporate the NHANES survey design. The shaded area is the pointwise 95% confidence band; the solid horizontal line at 1.0 denotes the null. The *P*-value for non-linearity is annotated in each panel. NHANES: National Health and Nutrition Examination Survey.

### Subgroup analyses

Because current asthma was the outcome associated with fiber, subgroup analyses focused on this outcome and are presented in Table 3. Point estimates were below unity in most strata and appeared lower in men than women, in adults aged 50 years or older than in younger adults, in normal-weight participants than in overweight or obese participants, and in never-smokers than in ever-smokers. However, none of the multiplicative interaction tests was statistically significant (sex *P*-interaction = 0.29; age *P*-interaction = 0.54; BMI-category *P*-interaction = 0.15; smoking *P*-interaction = 0.86). The subgroup-specific nominal *P* values were not adjusted for multiplicity and were not interpreted as evidence of differential effects. These patterns should therefore be regarded as exploratory and hypothesis-generating rather than evidence of true effect modification.

**Table 3:** Subgroup analyses of the association between Day-1 dietary fiber intake and current asthma

Subgroup	No.	OR (95% CI)	*P*	*P*-interaction
Overall				
All participants	6,145	0.80 (0.64, 0.99)	0.043	
Sex				
Male	2,982	0.69 (0.52, 0.93)	0.015	0.29
Female	3,163	0.87 (0.65, 1.15)	0.299	
Age group				
20–49 years	2,827	0.88 (0.72, 1.08)	0.200	0.54
≥50 years	3,318	0.67 (0.46, 0.96)	0.030	
Body mass index				
Normal (<25 kg/m^2^)	1,517	0.54 (0.38, 0.79)	0.002	0.15
Overweight (25–30)	1,932	1.01 (0.78, 1.30)	0.945	
Obese (≥30)	2,696	0.87 (0.65, 1.15)	0.310	
Smoking status				
Never smoker	3,540	0.68 (0.50, 0.93)	0.017	0.86
Ever smoker	2,605	1.08 (0.82, 1.43)	0.565	

Note. Effects are odds ratios with 95% CI from the fully adjusted model (Model 3). *P*-interaction is the *P*-value for the multiplicative fiber-by-subgroup product term. OR: odds ratio; CI: confidence intervals.

### Sensitivity analyses

The direction of the fiber-current-asthma association was generally stable across sensitivity analyses. Among 5,364 participants with two reliable recalls, mean 2-day fiber intake was 16.4 g/day (SD 8.2), the median was 14.2 g/day and the Day-1–Day-2 correlation was *r* = 0.44. In this separate repeated-recall analysis using the 2-day dietary weight, the OR was 0.74 per 1-standard-deviation of the 2-day mean (95% CI 0.61–0.90; *P* = 0.004). Because this analysis involved a smaller subset, WTDR2DPP rather than WTDRD1PP, and a different fiber standard deviation (8.2 vs. 9.8 g/day), the magnitude of this OR was not directly compared with the Day-1 OR; it was interpreted as directional consistency only. Two-day quartile estimates were also inverse, with ORs of 0.79 (95% CI 0.52–1.20), 0.68 (0.45–1.02) and 0.57 (0.36–0.90) for quartiles 2 through 4 versus quartile 1, respectively (*P* for trend = 0.019). Repeated-recall results are summarized in Supplementary Tables 3 and 4. Expressing fiber as density per 1,000 kcal yielded OR 0.80 (95% CI 0.66–0.97; *P* = 0.024) and is reported in Supplementary Table 5. Multiple imputation of missing covariates in the 7,302 otherwise eligible adults produced a similar Model 3 estimate (OR 0.81, 95% CI 0.66–0.99; *P* = 0.041), while the imputed BMI-adjusted estimate was OR 0.83 (95% CI 0.68–1.02; *P* = 0.078). Adding BMI in complete cases changed the Model 3 log-odds coefficient by approximately 11.1% (OR 0.80–0.82), indicating modest attenuation. The *E*-value was 1.81 for the Model 3 point estimate and 1.11 for the confidence limit closest to the null, indicating that relatively modest residual confounding could weaken the statistical evidence. Missing-data and BMI sensitivity results are summarized in Supplementary Table 5. Estimates after excluding current smokers (OR 0.80, 95% CI 0.62–1.03; *P* = 0.082) and participants with obesity (OR 0.77, 95% CI 0.58–1.02; *P* = 0.063) remained directionally similar. In the NHANES 2017–March 2020 subset with a history of asthma, fiber was not associated with asthma attacks (OR 1.21, 95% CI 0.84–1.74; *P* = 0.29). False-discovery-rate results for the three prespecified Model 3 outcomes are summarized in Supplementary Table 6.

## Discussion

In this nationally representative sample of U.S. adults, higher reported dietary fiber intake showed an inverse association with current asthma after adjustment for demographic, socioeconomic, lifestyle and clinical factors. The association was approximately monotonic and directionally stable across sensitivity analyses, including a separate repeated-recall analysis and a similar estimate after multiple imputation of missing covariates. However, the primary Model 3 association was marginal on the nominal scale and did not remain statistically significant after false-discovery-rate correction across the three prespecified asthma outcomes. The estimate was also modestly attenuated after BMI adjustment. In contrast, fiber intake was not associated with recent asthma attacks or asthma-related acute-care visits. Taken together, these findings support a cautious interpretation of dietary fiber as a correlate of current asthma prevalence rather than evidence of a causal protective effect.

The direction of our results is concordant with prior work, although the statistical evidence in this analysis is modest. The most directly comparable study, a previous NHANES analysis of chronic inflammatory airway disease, reported that higher fiber intake was associated with approximately 29% lower odds of asthma in the highest versus lowest intake category and described a linear inverse dose-response (21). Our quartile point estimate and spline pattern are directionally aligned with that report, while extending the question to the 2017–March 2020 pre-pandemic data and focusing specifically on asthma rather than a composite airway phenotype. The direction is also consistent with broader diet-asthma literature linking pro-inflammatory dietary patterns, lower diet quality, higher total energy intake and higher ultra-processed-food consumption to greater asthma prevalence, while plant-rich and Mediterranean-style patterns appear protective (14–19). Mendelian randomization analyses have also examined dietary exposures in relation to asthma susceptibility, offering complementary but not definitive evidence (20). The mean fiber intake in our sample was only about 16 g/day, well below recommended levels (6).

A coherent biological framework may account for an inverse fiber-asthma association centered on prevalent disease. Fermentable fiber that escapes small-intestinal digestion is metabolized by the colonic microbiota to short-chain fatty acids, which act locally and systemically through the gut-lung axis to calibrate pulmonary immunity (7, 8). Short-chain fatty acids, especially butyrate, promote regulatory T-cell differentiation and function, restrain type-2 innate lymphoid-cell and T-helper-2 cytokine responses central to allergic airway inflammation, and suppress pro-inflammatory signaling through free-fatty-acid receptors and histone-deacetylase inhibition; experimentally, they attenuate airway hyperreactivity and eosinophilic inflammation (9–11). Soluble fiber may also reinforce the intestinal barrier and reduce translocation of pro-inflammatory microbial products into the circulation, potentially dampening low-grade systemic inflammation (4, 5). These mechanisms are consistent with our baseline observation and prior analyses showing lower inflammatory biomarkers with higher fiber intake (30, 31). They may influence chronic airway inflammation more than acute, trigger-driven exacerbations, offering a plausible rationale for the dissociation observed between current asthma and acute events.

The BMI analysis was interpreted within a causal framework rather than as a simple confounder adjustment. Socioeconomic and lifestyle factors may act as common causes of both fiber intake and asthma, whereas dietary fiber may also influence adiposity and adiposity is itself related to asthma; BMI can therefore plausibly lie partly downstream of the exposure. For this reason, Model 3 without BMI was treated as the primary confounder-adjusted association and Model 4 as a sensitivity analysis rather than a definitive controlled direct effect. The requirement for measured BMI in the common complete-case sample was made solely to keep Models 1–4 on the same participants for direct coefficient comparison; it did not mean that BMI was treated as a Model 3 confounder. Adding BMI changed the log-odds coefficient by approximately 11.1%, from OR 0.80 to 0.82, consistent with modest attenuation rather than complete explanation by adiposity. At the same time, the *E*-value of 1.81 for the point estimate and 1.11 for the confidence limit closest to the null indicates limited robustness to residual unmeasured confounding. These results do not establish mediation by adiposity and should not be interpreted as evidence of a direct biological effect of fiber independent of BMI.

The subgroup findings are exploratory. Point estimates suggested potentially stronger inverse associations in men, older adults, normal-weight individuals and never-smokers, but all interaction tests were non-significant and subgroup-specific nominal *P*-values were not corrected for multiplicity. A lower point estimate in never-smokers could be biologically plausible if the oxidative and inflammatory stimulus of tobacco smoke obscures more modest dietary associations with airway inflammation, but the patterns by sex, age and BMI may equally reflect chance, differences in body composition, dietary reporting, or asthma phenotype across strata. These observations should therefore be treated as hypothesis-generating signals rather than evidence of genuine effect modification.

The null associations for asthma attacks and acute-care visits are important. Statistically, these outcomes were uncommon – 262 attacks and only 111 acute-care visits – so the analyses had limited power and wide CI, and a modest true association cannot be excluded. Conceptually, attacks and acute-care visits are downstream events among people with asthma and are driven by acute triggers, allergen and viral exposures, medication adherence and access to care – factors over which reported fiber intake would be expected to have limited short-term influence. It is therefore plausible that a dietary exposure related to chronic airway inflammation could be associated with asthma prevalence but not with acute exacerbations in a cross-sectional snapshot. This distinction is consistent with the null attack analysis among participants with a history of asthma and suggests that future studies should distinguish disease occurrence from disease control.

Reverse causation is an important alternative explanation for the observed current-asthma association. Current asthma reflects an existing disease state, whereas dietary fiber was measured contemporaneously, so the temporal ordering of exposure and outcome cannot be established. Adults with asthma may alter their diets because of respiratory symptoms, comorbid gastrointestinal conditions, weight-management advice, medication-related effects, or avoidance of foods perceived to provoke symptoms. Disease-related dietary changes could therefore generate an association between lower reported fiber intake and current asthma even in the absence of a protective causal effect of fiber. The present results should accordingly be interpreted as an association between reported fiber intake and asthma prevalence, not as evidence that increasing fiber prevents asthma.

This study has several strengths. It draws on a large, nationally representative sample of U.S. adults with standardized dietary, examination and questionnaire data, and all analyses incorporated the complex survey design with Taylor-linearized variance estimation. The simultaneous evaluation of prevalent asthma alongside two markers of acute disease activity, flexible spline characterization of the dose-response, repeated-recall analysis in 87.3% of the primary sample, explicit assessment of multiple outcome testing, staged treatment of BMI and missing-data sensitivity analyses using both participant comparisons and multiple imputation provide a transparent assessment of the stability and limitations of the observed association.

Several limitations tamper interpretation. Firstly, the cross-sectional design precludes temporal ordering and causal inference, and reverse causation remains plausible because participants with asthma may change their diet in response to disease, symptoms, comorbidity or clinical advice. Secondly, the primary exposure was based on a single Day-1 24-h recall and therefore remains susceptible to day-to-day variation and non-differential dietary measurement error. If such random error is approximately non-differential with respect to asthma status, it would generally be expected to attenuate an exposure-outcome association toward the null, although the direction of bias is not guaranteed in a multivariable observational model. The separate 2-day analysis was directionally consistent, but because it used a smaller subset, a different dietary weight and a different exposure standard deviation, the difference in OR magnitude cannot be attributed specifically to reduced random within-person error; two recalls also do not establish long-term usual intake. Thirdly, asthma status was questionnaire-based without spirometric or biomarker confirmation. Fourthly, residual and unmeasured confounding remains likely because high fiber intake clusters with health-promoting behaviors that are incompletely captured in NHANES; the modest *E*-values indicate that relatively weak residual confounding could move the estimate or its CI to the null. Fifthly, 15.8% of otherwise eligible participants were excluded from the common complete-case analyses because of missing BMI or model covariates, particularly income. This included 91 participants excluded solely because BMI was unavailable even though BMI was not included in the primary Model 3; BMI availability was required only to hold the sample constant across Models 1–4. This choice may have reduced precision and may contribute to selection bias. The excluded group differed modestly in fiber intake, education and smoking; although multiple imputation using all 7,302 otherwise eligible adults produced a similar estimate, selection bias cannot be fully excluded. Sixthly, the nominal current-asthma association did not remain statistically significant after false-discovery-rate correction across the three prespecified outcomes, and subgroup analyses were exploratory and unadjusted for multiplicity. Finally, we analyzed total dietary fiber rather than specific fiber types or food sources, did not measure the gut microbiome or short-chain fatty acids directly, and acute-event analyses were limited by small event counts. Findings therefore pertain to association patterns in non-institutionalized U.S. adults and require prospective confirmation.

## Conclusions

In a nationally representative sample of U.S. adults, higher reported dietary fiber intake showed an inverse association with current asthma, with an approximately monotonic dose-response and directionally consistent estimates in repeated-recall and multiple-imputation analyses, but no association with recent asthma attacks or asthma-related acute-care visits. The primary current-asthma result was statistically modest and did not remain significant after false-discovery-rate correction across the three prespecified outcomes, while BMI adjustment and *E*-value analysis further underscored uncertainty about causal interpretation. Dietary fiber should therefore be regarded as a plausible correlate of lower asthma prevalence rather than a demonstrated protective factor. Prospective cohorts and randomized trials that establish temporal ordering, characterize long-term fiber intake and source, and measure microbiome and inflammatory intermediates are needed to determine whether increasing dietary fiber can influence asthma risk or burden.

## Supplementary Material



## Data Availability

The authors have nothing to report.

## Declarations

Human ethics and consent to participate declarations An ethics statement is not applicable because this study is based exclusively on published literature.
