# Supplementary material for: Association of dietary fiber intake with current asthma and recent asthma outcomes: a cross-sectional analysis of National Health and Nutrition Examination Survey 2017–March 2020 pre-pandemic data
Source: Food Nutr Res. 2026 Oct 23;70:10.29219/fnr.v70.14782. doi: 10.29219/fnr.v70.14782 (PMC13732517; doi:10.29219/fnr.v70.14782)
Supplement: Supplementary file 1 [file FNR-70-14782-s1.docx]

Table S1. Comparison of the common complete-case analytic sample and otherwise eligible participants excluded because of missing BMI or model covariates.

| **Characteristic** | **Complete cases N = 6,145** | **Excluded for missing BMI/covariates N = 1,157** |
| --- | --- | --- |
| Age, years | 48.7 ± 17.4 | 46.9 ± 18.2 |
| Female, % | 51.6 | 53.8 |
| Dietary fiber, g/day | 16.2 ± 9.8 | 15.3 ± 9.4 |
| Current asthma, % | 8.7 | 9.4 |
| BMI, kg/m² | 29.8 ± 7.1 | 30.2 ± 7.5* |
| Non-Hispanic White, % | 64.8 | 59.5 |
| Non-Hispanic Black, % | 10.6 | 13.7 |
| Some college or higher, % | 63.0 | 55.6 |
| Current smoker, % | 16.2 | 20.5 |
| Hypertension, % | 33.4 | 32.1 |
| Diabetes, % | 11.6 | 11.9 |

**Note:** Values are weighted means ± standard deviations or weighted percentages unless otherwise indicated. The common complete-case sample required all variables needed across Models 1-4 so that staged estimates could be compared in the same participants; therefore measured BMI was required for sample membership even though BMI was not included as a covariate in the primary Model 3. *BMI summary among excluded participants with available BMI.

Table S2. Extent of missingness in BMI and model covariates among otherwise eligible participants used to define the common complete-case sample.

| **Variable** | **Missing n** | **Missing %** |
| --- | --- | --- |
| BMI | 91 | 1.2% of 7,302 |
| Family income-to-poverty ratio | 887 | 12.3% of 7,211 |
| Physical activity | 153 | 2.1% of 7,211 |
| Alcohol status | 61 | 0.8% of 7,211 |
| Smoking status | 20 | 0.3% of 7,211 |
| Education | 12 | 0.2% of 7,211 |
| Other clinical covariates | <20 each | <0.3% each of 7,211 |

**Note:** Among 7,302 otherwise eligible adults, 91 had missing BMI and were excluded from the common complete-case sample solely to preserve the same participants across Models 1-4. The denominator for non-BMI covariates is the 7,211 otherwise eligible participants with measured BMI. Missingness can overlap across covariates, so counts do not sum to the number excluded for missing covariate data.

Table S3. Separate Day-1 primary and two-day repeated-recall sensitivity analyses for current asthma.

| **Measure** | **Day-1 primary analysis** | **Two-day mean sensitivity analysis** |
| --- | --- | --- |
| N | 6,145 | 5,364 |
| Proportion of primary sample | 100% | 87.3% |
| Fiber mean, g/day | 16.2 ± 9.8 | 16.4 ± 8.2 |
| Median, g/day | 13.8 | 14.2 |
| Q1 cutoff, g/day | 8.9 | 9.6 |
| Q2 cutoff, g/day | 13.8 | 14.2 |
| Q3 cutoff, g/day | 20.4 | 20.1 |
| Day-1-Day-2 correlation | — | r = 0.44 |
| OR per 1-SD | 0.80 | 0.74 |
| 95% CI | 0.64-0.99 | 0.61-0.90 |
| P value | 0.043 | 0.004 |

**Note:** The Day-1 analysis used WTDRD1PP and the two-day analysis used WTDR2DPP. These columns are separate analyses rather than a paired effect-size comparison: they differ in participant subset, dietary weight and exposure standard deviation (9.8 versus 8.2 g/day). Accordingly, the OR magnitudes should not be directly compared or interpreted as quantifying attenuation from random measurement error. Averaging two recalls can reduce the influence of day-to-day variation, but two recalls do not establish long-term usual intake.

Table S4. Two-day mean fiber quartiles and current asthma

| **Two-day mean fiber** | **OR (95% CI)** | **P value** |
| --- | --- | --- |
| Q1 | 1.00 (reference) | — |
| Q2 | 0.79 (0.52-1.20) | 0.26 |
| Q3 | 0.68 (0.45-1.02) | 0.063 |
| Q4 | 0.57 (0.36-0.90) | 0.017 |
| P for trend | — | 0.019 |

Table S5. Sensitivity analyses for the association between dietary fiber and current asthma.

| **Analysis** | **N** | **Odds ratio** | **95% CI** | **P value** | **Additional result** |
| --- | --- | --- | --- | --- | --- |
| Fiber-density Model 3 (per 1-SD density) | 6,145 | 0.80 | 0.66-0.97 | 0.024 | Fiber expressed per 1,000 kcal |
| Complete-case Model 3 | 6,145 | 0.80 | 0.64-0.99 | 0.043 | E-value = 1.81; CI-limit E-value = 1.11 |
| Multiple-imputation Model 3 | 7,302 | 0.81 | 0.66-0.99 | 0.041 | Estimate closely matched complete-case result |
| Complete-case Model 4 + BMI | 6,145 | 0.82 | 0.66-1.02 | 0.076 | 11.1% attenuation of log-odds coefficient |
| Multiple-imputation Model 4 + BMI | 7,302 | 0.83 | 0.68-1.02 | 0.078 | Estimate closely matched complete-case result |

Note: Odds ratios are per 1-SD increase in the specified fiber exposure. Multiple imputation used chained equations with 20 imputed datasets and Rubin's rules and included all 7,302 otherwise eligible adults. Model 3 excluded BMI from the core confounder set; Model 4 added BMI as a sensitivity analysis. The complete-case rows use the common N = 6,145 sample required across Models 1-4 for direct coefficient comparison; BMI availability was therefore required for sample membership even though BMI was not a Model 3 covariate. E-values quantify the minimum strength of association that an unmeasured confounder would need with both exposure and outcome, conditional on measured covariates, to explain the observed point estimate or move the confidence interval to the null.

Table S6. Benjamini-Hochberg false-discovery-rate correction across the three prespecified Model 3 asthma outcomes.

| **Outcome** | **Nominal P value** | **BH-FDR q value** |
| --- | --- | --- |
| Current asthma | 0.043 | 0.129 |
| Asthma attack, past 12 months | 0.523 | 0.631 |
| Asthma-related acute-care visit | 0.631 | 0.631 |

Note: The three prespecified asthma outcomes were treated as one hypothesis family. Progressive adjustment stages within the same outcome were not treated as independent hypotheses.
